# Widening the Spectrum of Risk Factors, Comorbidities, and Prodromal Features of Parkinson Disease

**DOI:** 10.1001/jamaneurol.2022.3902

**Published:** 2022-11-07

**Authors:** Anette Schrag, Jens Bohlken, Lotte Dammertz, Stefan Teipel, Wiebke Hermann, Manas K. Akmatov, Jörg Bätzing, Jakob Holstiege

**Affiliations:** 1 Department of Clinical and Movement Neurosciences, University College London, London, United Kingdom; 2 Institut für Sozialmedizin, Arbeitsmedizin und Public Health der Medizinischen Fakultät der Universität Leipzig, Leipzig, Germany; 3 Central Research Institute of Ambulatory Health Care in Germany, Department of Epidemiology and Healthcare Atlas, Berlin, Germany; 4 Deutsches Zentrum für Neurodegenerative Erkrankungen Rostock/Greifswald, Rostock, Germany; 5 Department of Psychosomatic Medicine, University of Rostock, Rostock, Germany; 6 Department of Neurology, University of Rostock, Rostock, Germany

## Abstract

**Importance:**

The prodromal phase of Parkinson disease (PD) may last for more than 10 years. Recognition of the spectrum and occurrence of risk factors, comorbidities, and prodromal features of PD can increase understanding of the causes and development of the disease and help identify individuals at risk.

**Objective:**

To identify the association of a subsequent diagnosis of PD with a range of risk factors and prodromal features, including lifestyle factors, comorbidities, and potential extracerebral manifestations of PD.

**Design, Setting, and Participants:**

This was a case-control study using insurance claims of outpatient consultations of patients with German statutory health insurance between January 1, 2011, and December 31, 2020. Included were patients with incident diagnosis of PD without a previous diagnosis of parkinsonism or dementia and controls matched 1:2 for age, sex, region, and earliest year of outpatient encounter.

**Exposures:**

Exposures were selected based on previous systematic reviews, case-control and cohort studies reporting on risk factors, comorbidities, and prodromal features of PD.

**Main Outcomes and Measures:**

Previously postulated risk factors and prodromal features of PD, using the *International Statistical Classification of Diseases and Related Health Problems, Tenth Revision (ICD-10)* coding.

**Results:**

A total of 138 345 patients with incident PD (mean [SD] age, 75.1 [9.8] years; 73 720 male [53.3%]) and 276 690 matched controls (mean [SD] age, 75.1 (9.8) years; 147 440 male [53.3%]) were identified. Study participants were followed up for a mean (SD) of 6.0 (2.0) years. Consistent with previous reports, risk factors and prodromal features associated with PD included traumatic brain injury, odds ratio (OR), 1.62; 95% CI, 1.36-1.92; alcohol misuse, OR, 1.32; 95% CI, 1.21-1.44; hypertension, OR, 1.29; 95% CI, 1.26-1.31; anosmia, OR, 2.16; 95% CI, 1.59-2.93; and parasomnias (including RBD), OR, 1.62; 95% CI, 1.42-1.84. In addition, there were associations with restless legs syndrome (OR, 4.19; 95% CI, 3.91-4.50), sleep apnea (OR, 1.45; 95% CI, 1.37-1.54), epilepsy (OR, 2.26; 95% CI, 2.07-2.46), migraine (OR, 1.21; 95% CI, 1.12-1.29), bipolar disorder (OR, 3.81; 95% CI, 3.11-4.67), and schizophrenia (OR, 4.48; 95% CI, 3.82-5.25). The following diagnoses were also found to be associated with PD: sensory impairments beyond anosmia, such as hearing loss (OR, 1.14; 95% CI, 1.09-1.20) and changes of skin sensation (OR, 1.31; 95% CI, 1.21-1.43). There were also positive associations with skin disorders (eg, seborrheic dermatitis, OR, 1.30; 95% CI, 1.15-1.46; psoriasis, OR, 1.13; 95% CI, 1.05-1.21), gastrointestinal disorders (eg, gastroesophageal reflux, OR, 1.29; 95% CI, 1.25-1.33; gastritis, OR, 1.28; 95% CI, 1.24-1.33), conditions with a potential inflammatory component (eg, seronegative osteoarthritis, OR, 1.21; 95% CI, 1.03-1.43), and diabetes types 1 (OR, 1.32; 95% CI, 1.21-1.43) and 2 (OR, 1.24; 95% CI, 1.20-1.27). Associations even 5 to 10 years before diagnosis included tremor (odds ratio [OR], 4.49; 95% CI, 3.98-5.06), restless legs syndrome (OR, 3.73; 95% CI, 3.39-4.09), bipolar disorder (OR, 3.80; 95% CI, 2.82-5.14), and schizophrenia (OR, 4.00; 95% CI, 3.31-4.85).

**Conclusions and Relevance:**

Results of this case-control study suggest that the associations found between PD and certain risk factors, comorbidities, and prodromal symptoms in a representative population may reflect possible early extrastriatal and extracerebral pathology of PD. This may be due to shared genetic risk with PD, medication exposure, or direct causation, or represent pathophysiologically relevant factors contributing to the pathogenesis of PD.

## Key Points

**Question:**

What risk factors, comorbidities, and prodromal symptoms preceded the diagnosis of Parkinson disease (PD) in a large representative routine-care database?

**Findings:**

In this case-control study of 138 345 patients with incident PD and 276 690 matched controls, an increased risk of PD was associated with a range of risk factors, comorbidities, and prodromal features, particularly tremor, restless legs syndrome, and both schizophrenia and bipolar disorder; comorbidities such as diabetes types 1 and 2, epilepsy, sensory skin disturbances, and gastrointestinal disorders; and risk factors such as alcohol misuse and traumatic head injury.

**Meaning:**

These associations may reflect possible early extrastriatal and extracerebral pathology of PD; risk factors due to shared genetic risk with PD, medication exposure, or direct causation; or may represent pathophysiologically relevant factors contributing to the pathogenesis of PD.

## Teaser

This case-control study investigates the risk factors, comorbidities, and prodromal symptoms associated with a diagnosis of Parkinson disease in a large representative database.

## Introduction

Prodromal features of Parkinson disease (PD) can start more than a decade before the typical clinical symptoms allow a diagnosis.^1,2^ In addition, there is increasing evidence for a number of possible risk factors that may predispose to the manifestation of the disease or facilitate development or spread of pathological lesions. These risk factors include well-known genetic or environmental risk factors but also diabetes type 2 or gastric pathology, which may increase spread of pathology from the enteric nervous system via the vagal nerve to the central nervous system.^3,4^ The recognition of such risk factors and prodromal features of PD together with the presence of Lewy body pathology in peripheral organs and early extrastriatal brain pathology several years before PD diagnosis have widened our understanding of the development of the disease. Specifically, these findings suggest that disease onset may not only occur in the brain but also in gastrointestinal and other extracerebral systems.^5,6^ These insights have also offered the opportunity to explore early biomarkers and mechanisms of pathogenesis. To date, the best-established prodromal features are subtle motor symptoms, rapid eye movement sleep behavior disorder (RBD; a rare but highly specific condition),^7,8^ hyposmia/anosmia (a common and relatively nonspecific feature),^9,10^ neuropsychiatric manifestations (eg, depression and anxiety), autonomic features (eg, constipation and urinary and sexual dysfunction), dizziness and fatigue, and pain.^1^ However, other prodromal features have been suggested but with little or divergent evidence. Some may reflect striatal or extrastriatal involvement like restless legs syndrome^11,12^ and cognitive changes^13^ or early deposition of α-synuclein aggregates in peripheral tissues, including skin.^14,15,16,17^ Several studies have suggested that infections with cytomegalovirus or Epstein-Barr virus may predate the diagnosis of PD and may represent triggers, risk factors, or causes of the onset of PD.^18,19,20,21^ Additional associations with potential risk factors include lack of a smoking history, a family history of PD, tremor, or head trauma.^4^ Associations are less consistent or divergent with dietary factors,^22^ alcohol intake,^23,24,25^ cholesterol levels,^26,27,28^ and hypertension^4,29^ as well as with type 2 diabetes,^30,31,32^ osteoarthritis, and inflammatory bowel disease.^33,34,35^ Finally, other studies have suggested associations with schizophrenia,^36,37^ bipolar disorder,^38,39^ epilepsy,^40,41^ and migraine.^42,43,44^ Although some studies indicate that the association with schizophrenia prevails even when excluding drug-induced parkinsonism,^36,37^ at least part of the associations with these diseases may be due to medications known to be associated with drug-induced parkinsonism.

Most studies to date include relatively small sample sizes that may have missed subtle associations, included a limited number of exposures precluding comparisons in terms of strength and timeline of association, or are retrospective studies and limited by recall bias. Availability of large data sets, collected in routine care, enables the detection and comparison of subtle associations of multiple risk factors, which may otherwise not be identified. Here, we used a routine-care database comprising insurance claims of outpatient consultations in the German statutory health insurance (covers 87% of all inhabitants of Germany) to analyze data over a 10-year period.

## Methods

### Study Design

This was a case-control study using insurance claims of outpatient consultations of patients with German statutory health insurance and incident PD identified between January 1, 2011, and December 31, 2020, using general and specialist practice data from a source population of 72 842 190 people in 2020.^45^ The use of claims data for scientific research in Germany is regulated by the Code of Social Law (Sozialgesetzbuch, SGB V). Ethical approval and informed consent are not required for routinely collected pseudonymized data. This study followed the Strengthening the Reporting of Observational Studies in Epidemiology (STROBE) reporting guidelines.

Patients cared for by more than 1 medical professional were only included once. Individuals were included if at least 3 years of outpatient data before diagnosis of PD or index date were available, in order to limit the possibility of including patients with a previous diagnosis of PD that was first recorded by a new treating physician during the patient registration period. Thus, cases of newly diagnosed PD and controls were identified in the data set from January 1, 2014, to December 31, 2020, if they attended 1 or more outpatient visits in the respective year and also received outpatient services at least 1 time 3 years before the index year or earlier. Diagnosis of PD was defined as the presence of an *International Statistical Classification of Diseases and Related Health Problems, Tenth Revision (ICD-10) *diagnostic code (*ICD-10*: G20) in more than 1 insurance claim period (3 months) without a previous diagnosis of parkinsonism (*ICD-10*: G20, G21, or G22) in the preceding 3 years. Patients and controls with a diagnosis of dementia (*ICD-10*: F03, F00) within the 3 years before the index date were excluded. We matched cases to controls (1:2) without a diagnosis of PD (*ICD-10*: G20, G21, or G22) in the index year or the preceding 3 years, with an index date within the same 3-month time period as the case’s PD diagnosis, and matched for age, sex, geographic region of residence, and earliest year of outpatient encounter within the study period.

Data on the presence of defined diagnoses with a potential association with subsequent diagnosis of PD, identified from a review of the literature, were then extracted for each individual from general practice data, both for each year and grouped for the periods less than 1 year, 2 to 4 years, and 5 to 10 years before index date, independent of calendar year and first onset. The time slicing was oriented on previous studies.^1^ *ICD* codes for potential prodromal features, risk factors, and comorbidities were defined as described in eTable 1 in the Supplement. This list originated from the literature review and discussion with PD experts. Only prodromal features, risk factors, and comorbidities coded by general practitioners were included in this analysis.

### Statistical Analysis

Odds ratios (ORs) were calculated for potential prodromal features of PD in the year before index date and pooled for the periods 2 to 4 years and 5 to 10 years before index date. The 95% CIs were calculated using the method by Altmann^46^ with conservative Bonferroni adjustment for multiple comparisons. Statistical significance was assumed when the 95% CI of the OR did not overlap the null value (eg, OR = 1.0). Statistical analyses were performed using SAS, version 9.4 (SAS Institute).

## Results

A total of 138 345 patients with incident PD (mean [SD] age, 75.1 [9.8] years; 73 720 male [53.3%]; 64 625 female [46.7%]) in the period between 2014 and 2020 and 276 690 matched controls (mean [SD] age, 75.1 (9.8) years; 147 440 male [53.3%]; 129 250 female [46.7%]) were identified. Their demographic characteristics for each time period are given in the Table. Mean (SD) follow-up time was 6.0 (2.0) years in both cases and controls. A total of 102 360 patients (74%) with PD and 27 652 controls (10%) were examined by a neurologist during the insurance quarter of diagnosis. The following presentation of the results is grouped according to the role of a factor as possible prodrome of disease or as risk or comorbid factor.

**Table.** Characteristics of Patients With Incident Parkinson Disease and Controls

Variable	Total		Retrospective data					
			With 1 y		With 2-4 y		With 5-10 y	
	Cases	Controls	Cases	Controls	Cases	Controls	Cases	Controls
No.	138 345	276 690	138 345	276 690	138 345	276 690	106 957	213 914
Sex, No. (%)								
Female	64 625 (46.7)	129 250 (46.7)	64 625 (46.7)	129 250 (46.7)	64 625 (46.7)	129 250 (46.7)	49 656 (46.4)	99 312 (46.4)
Male	73 720 (53.3)	147 440 (53.3)	73 720 (53.3)	147 440 (53.3)	73 720 (53.3)	147 440 (53.3)	57 301 (53.6)	114 602 (53.6)
Age at index date, mean (SD) [range], y	75.1 (9.8) [40-105]	75.1 (9.8) [40-105]	75.1 (9.8) [40-105]	75.1 (9.8) [40-105]	75.1 (9.8) [40-105]	75.1 (9.8) [40-105]	75.14 (9.8) [40-105]	75.1 (9.8) [40-104]
Follow-up time, mean (SD), y^a^	6.0 (2.0)	6.0 (2.0)	6.0 (2.0)	6.0 (2.0)	6.0 (2.0)	6.0 (2.0)	6.7 (1.6)	6.7 (1.6)

^a^ Time from first recorded outpatient visit during observation period to index date.

### Suspected Prodromal Presentations of PD

**Figure 1.**
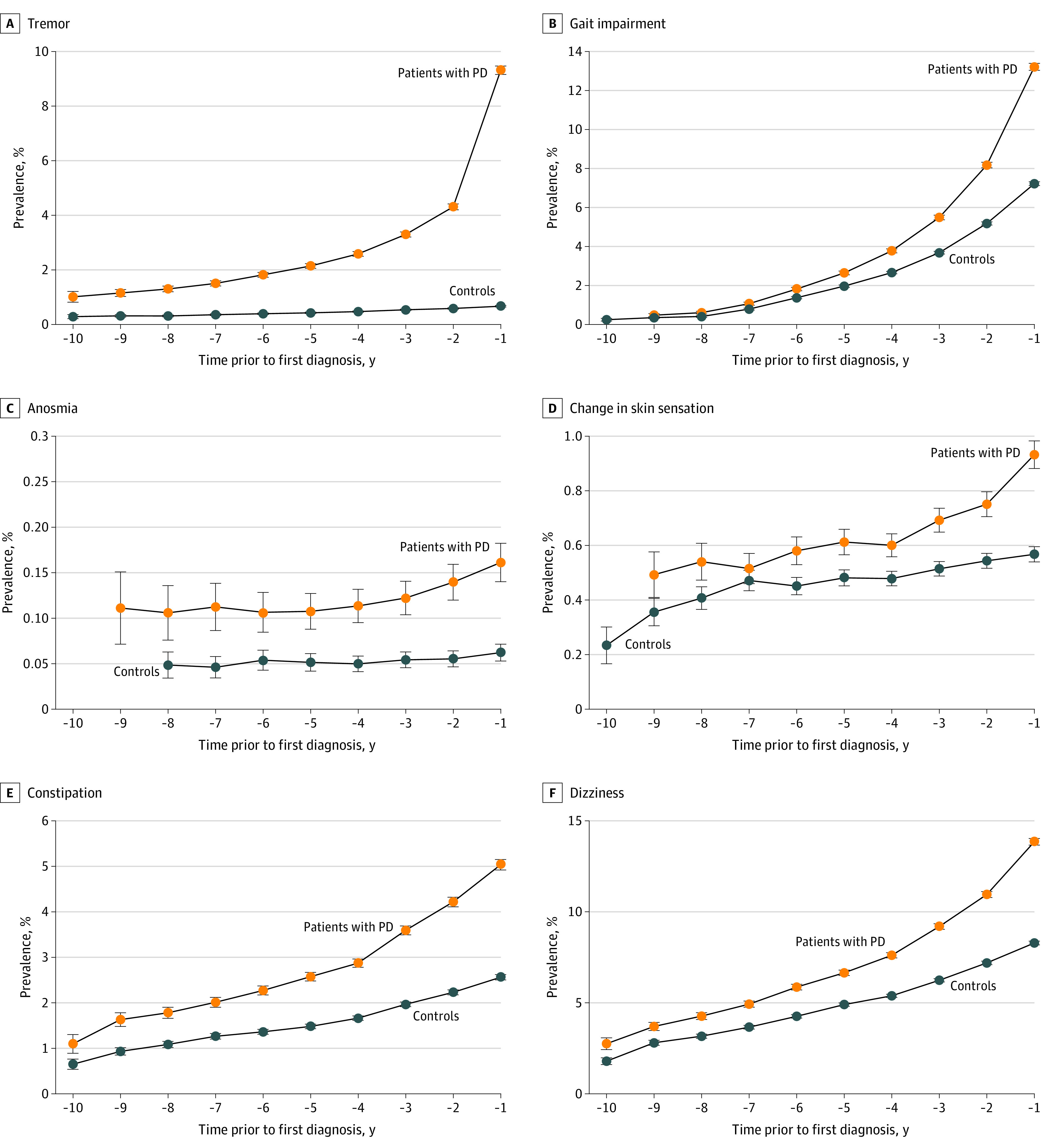
Prevalence of Motor, Sensory, and Autonomic Presentations Most Strongly Associated With Parkinson Disease (PD) by Year Before Diagnosis Compared With Controls Prevalence of tremor (A), gait impairment (B), anosmia (C), skin sensation (D), constipation (E), and dizziness (F) associated with PD by year before diagnosis.

**Figure 2.**
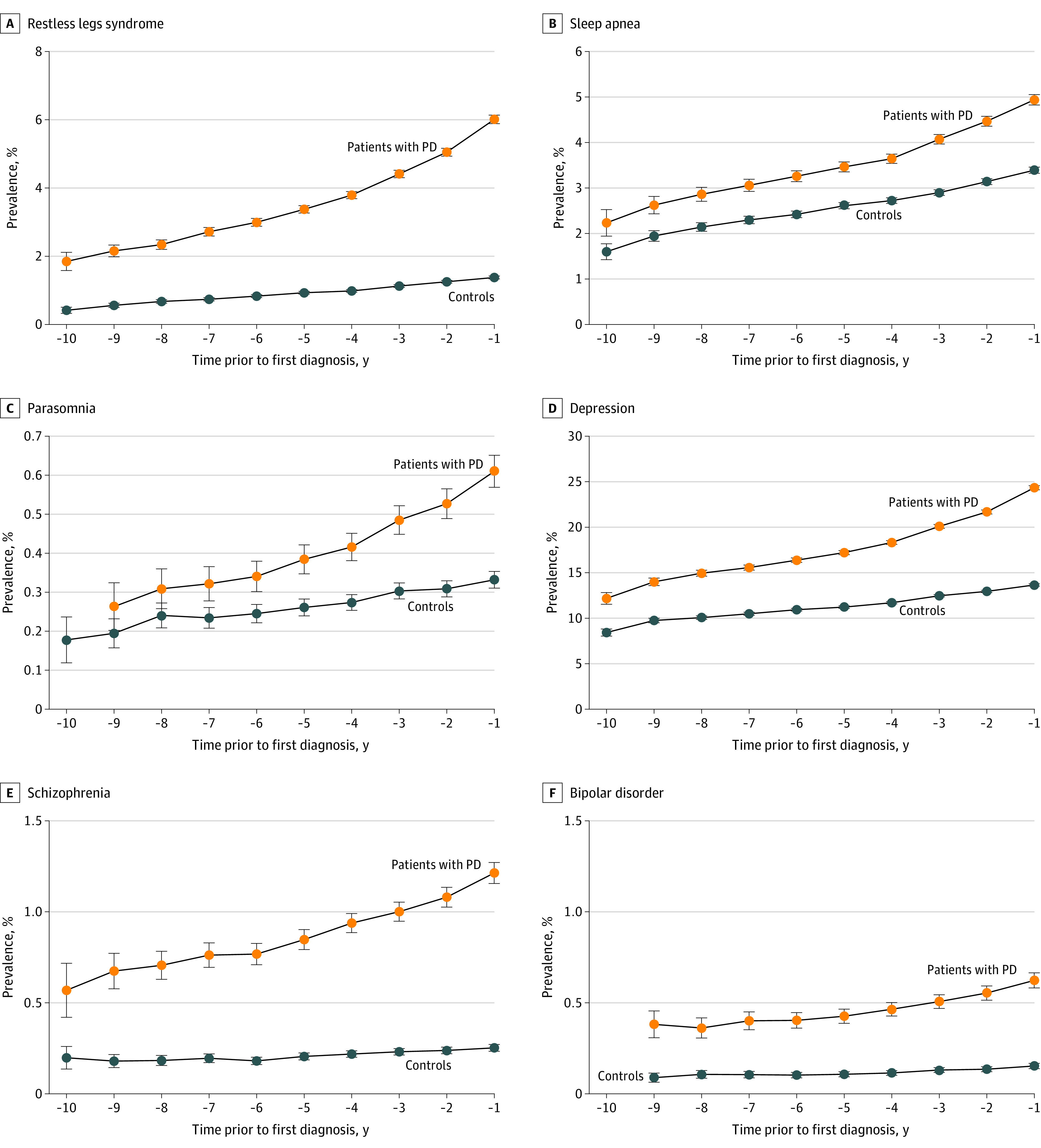
Prevalence of Sleep and Psychiatric Presentations Associated With Parkinson Disease (PD) by Year Before Diagnosis Compared With Controls Prevalence of restless legs syndrome (A), sleep apnea (B), parasomnia (C), depression (D), schizophrenia (E), and bipolar disorder (F) associated with PD by year before diagnosis.

**Figure 3.**
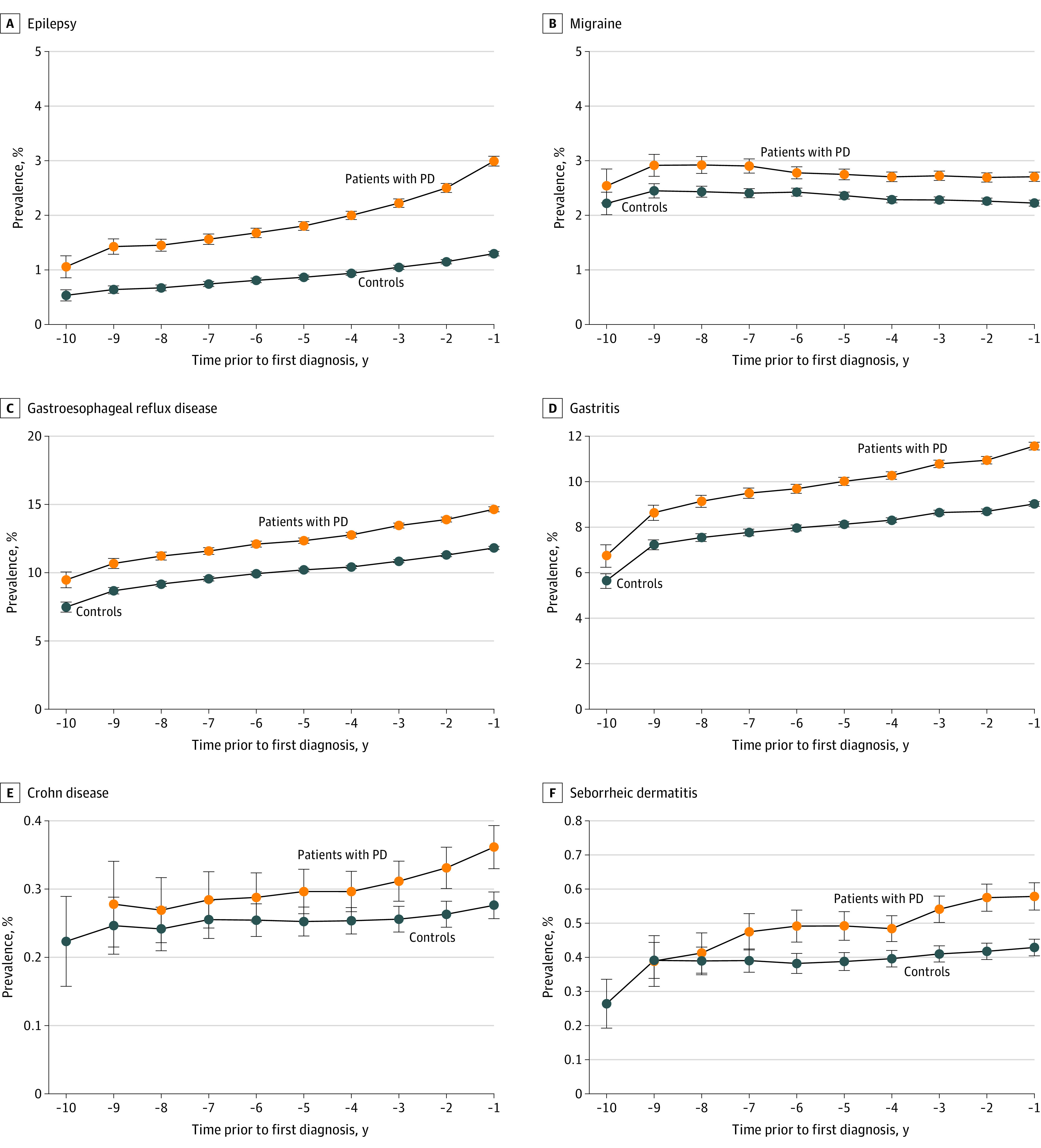
Prevalence of Some Comorbidities Associated With Parkinson Disease (PD) by Year Before Diagnosis Compared With Controls Prevalence of epilepsy (A), migraine (B), gastroesophageal reflux disease (C), gastritis (D), Crohn disease (E), and seborrheic dermatitis (F) associated with PD by year before diagnosis.

There were positive associations for the overall observation period with a subsequent diagnosis of PD for the motor features of tremor (OR, 11.38; 95% CI, 10.51-12.32), gait impairment (OR, 1.90; 95% CI, 1.83-1.98) (Figure 1), stiffness of joints (OR, 1.32; 95% CI, 1.17-1.50), shoulder pain (OR, 1.15; 95% CI, 1.06-1.24), and neck pain (OR, 1.16; 95% CI, 1.12-1.20) (eFigure in the Supplement). The autonomic presentations of dizziness (OR, 1.60; 95% CI, 1.55-1.66), postural hypotension (OR, 1.40; 95% CI, 1.32-1.49), constipation (OR, 1.84; 95% CI, 1.76-1.93), features of sexual dysfunction (OR, 1.20; 95% CI, 1.11-1.30), and neurogenic bladder (OR, 1.72; 95% CI, 1.52-1.94) also revealed positive associations with a diagnosis of PD. In addition, there were associations between the following features and PD: fatigue (OR, 1.43; 95% CI, 1.37-1.50); the neuropsychiatric presentations of depression (OR, 1.86; 95% CI, 1.81-1.92) (Figure 2), anxiety (OR, 1.65; 95% CI, 1.57-1.74), and memory problems (OR, 1.72; 95% CI, 1.59-1.85); the sleep disorders of restless leg syndrome (OR, 4.19; 95% CI, 3.91-4.50), parasomnias (including RBD; OR, 1.62; 95% CI, 1.42-1.84), sleep apnea (OR, 1.45; 95% CI, 1.37-1.54), insomnia (OR, 1.40; 95% C,I 1.31-1.49), other sleep disorders (OR, 1.41; 95% CI, 1.35-1.47), and, although rare, hypersomnia (OR, 2.16; 95% CI, 1.27-3.68) (eTable 3 in the Supplement). Further, for sensory changes including anosmia (OR, 2.16; 95% CI, 1.59-2.93), hearing loss (OR, 1.14; 95% CI, 1.09-1.20), alterations in skin sensation (OR, 1.31; 95% CI, 1.21-1.43), nonspecific pain (OR, 1.13; 95% CI, 1.09-1.17), and subjective visual disturbance (OR, 1.26; 95% CI, 1.01-1.57) and for diagnoses of the skin conditions seborrheic dermatitis (OR, 1.30; 95% CI, 1.15-1.46) (Figure 3), psoriasis (OR, 1.13; 95% CI, 1.05-1.21), and dermatophytosis (OR, 1.25; 95% CI, 1.19-1.32), there were positive associations with a diagnosis of PD.

### Association With Suspected Risk Factors and Comorbidities

**Figure 4.**
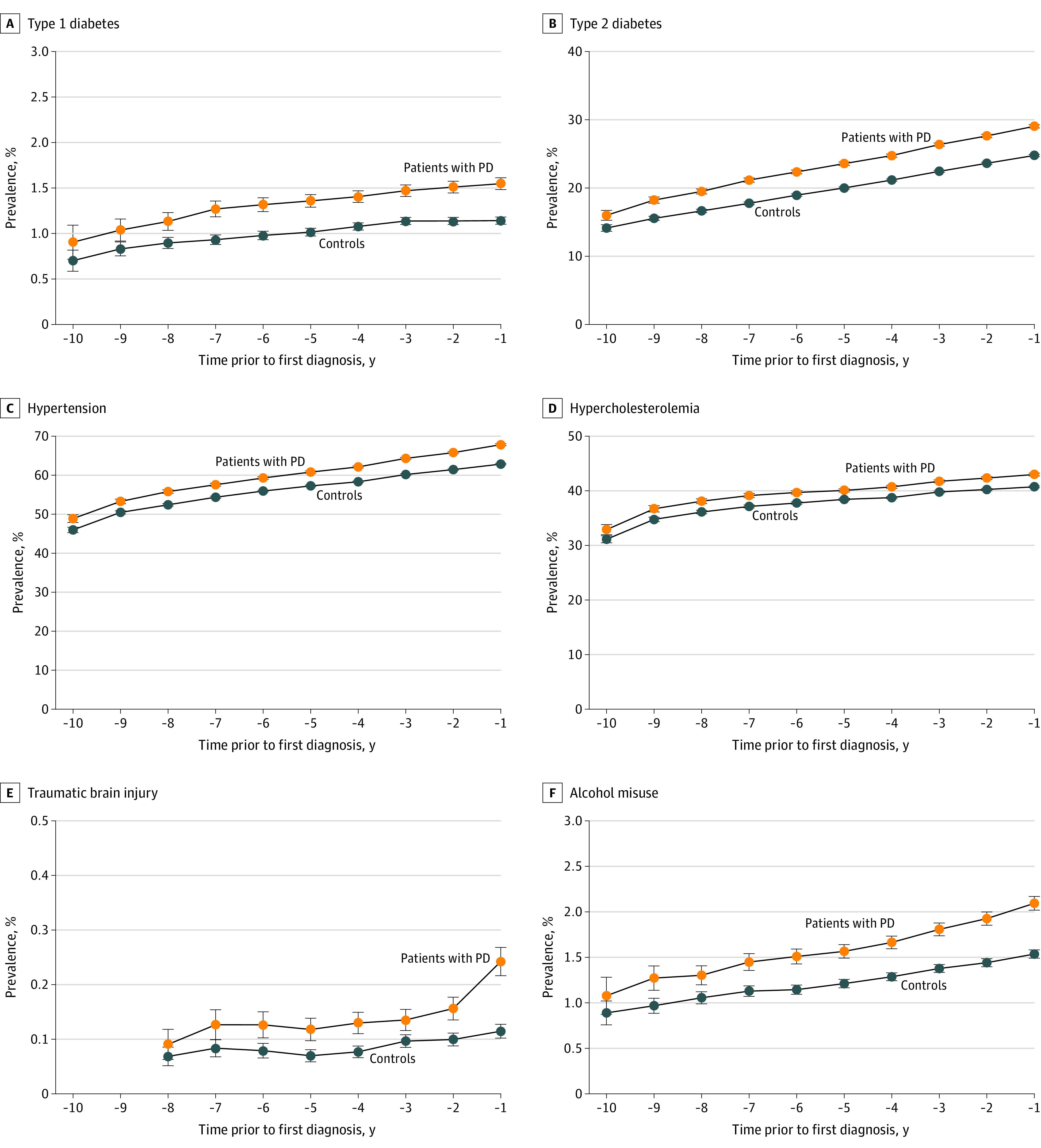
Prevalence of Other Risk Factors Associated With Parkinson Disease (PD) by Year Before Diagnosis Compared With Controls Prevalence of type 1 diabetes (A), type 2 diabetes (B), hypertension (C), hypercholesterolemia (D), traumatic brain injury (E), and alcohol misuse (F) with 95% CI error bars for each year before diagnosis of PD.

There was an increased OR for preceding alcohol misuse (OR, 1.32; 95% CI, 1.21-1.44) and traumatic brain injury (OR, 1.62; 95% CI, 1.36-1.92) as well as for hypertension (OR, 1.29; 95% CI, 1.26-1.31) and hypercholesterinemia (OR, 1.11; 95% CI, 1.08-1.13) (Figure 4). However, there was a reduced OR for nicotine misuse (OR, 0.92; 95% CI, 0.86-0.98) with PD. In addition, both diabetes type 1 (OR, 1.32; 95% CI, 1.21-1.43) and type 2 (OR, 1.24; 95% CI, 1.20-1.27) were associated with a subsequent diagnosis of PD overall and in all time periods before diagnosis of PD (eTable 2 in the Supplement; Figure 1).

Associations for comorbidities with PD were found for the diagnoses of schizophrenia (OR, 4.48; 95% CI, 3.82-5.25) and bipolar disorder (OR, 3.81; 95% CI, 3.11-4.67), with increased ORs also for epilepsy (OR, 2.26; 95% CI, 2.07-2.46), migraine (OR, 1.21; 95% CI, 1.12-1.29), osteoarthritis (OR, 1.20; 95% CI, 1.17-1.23), seropositive inflammatory arthritis (OR, 1.21; 95% CI, 1.03-1.43), and other inflammatory arthritis (OR, 1.19; 95% CI, 1.11-1.27). There was also an increased OR for the gastrointestinal comorbidities of gastroesophageal reflux disease (OR, 1.29; 95% CI, 1.25-1.33), gastritis (OR, 1.28; 95% CI, 1.24-1.33), and gastric ulcer (OR, 1.24; 95% CI, 1.12-1.37), with less-consistent associations over time periods for duodenal ulcer (OR, 1.13; 95% CI, 1.00-1.29), Crohn disease (OR, 1.21; 95% CI, 0.99-1.48), and ulcerative colitis (OR, 1.23; 95% CI, 1.06-1.43). There was no significant association in any time period for gastrojejunal ulcer (OR, 1.25; 95% CI, 0.81-1.92) and peptic ulcer (OR, 1.34; 95% CI, 0.97-1.86). There was no significant association for cytomegaloviral disease (OR, 1.05; 95% CI, 0.61-1.79) and infectious mononucleosis (OR, 1.46; 95% CI, 0.94-2.25), but these were rare.

## Discussion

In this large, representative, case-control study of PD based on claims data, we found a number of previously known early features and a range of previously unreported or controversial associations with subsequent diagnosis of PD. Among the early motor features, there were associations observed for tremor, which had a relatively high prevalence in those with a subsequent diagnosis of PD but rarely occurred in the control population (<1%). Changes in gait were common in both the PD and the control population but, together with shoulder pain and neck pain, were already increased 5 years before diagnosis, whereas detection of joint stiffness as a marker of rigidity was relatively uncommon before diagnosis. Consistent with previous reports,^1^ we found associations with neuropsychiatric features of early and prodromal PD, including depression and less commonly, anxiety,^1^ notably even in the earliest prediagnostic period. Interestingly, these neuropsychiatric features included memory complaints even more than 5 years before diagnosis, albeit much less commonly than depression or anxiety. Among the autonomic features, dizziness was present in more than 10% of patients more than 5 years before diagnosis of PD. Hypotension was relatively rare overall but more frequent in subsequent PD cases than in controls in all time periods. Possible interactions of hypotension with medication could not be assessed with our data. Constipation was only present in a relatively small proportion of patients before diagnosis of PD in this study, which was lower than in previous studies^1,2^ and may be due to underreporting. Sexual dysfunction and symptoms of neurogenic bladder disturbances had a low prevalence but were more frequently reported than in controls across all time periods. All sleep disorders were more common in the group with subsequent PD than in controls, including diagnostic codes used for parasomnias. This diagnostic code also covers RBD for which no specific code was available. However, other sleep disturbances, including insomnia, were also more commonly diagnosed before PD diagnosis as previously reported.^1,47^ RBD is thought to affect approximately 1% of the general population,^48^ but the condition is probably undiagnosed in the majority of patients because symptoms of RBD or other sleep disturbances are often underreported and undervalued in routine care. Furthermore, it is possible that diagnoses of sleep disorders, including parasomnias, nightmares, and insomnia, reflect underlying RBD, which would require specific questioning and polysomnography for a definite diagnosis. Sleep apnea has also been reported to be increased in patients with PD and been associated with risk of subsequent PD.^49,50^ Although information on diagnostic test results was not available, our study results also suggested an associated increased risk of a clinical diagnosis of sleep apnea in cases with a subsequent diagnosis of PD. Hypersomnia, although more common in those with subsequent diagnosis of PD, was not frequently diagnosed. This may have been due to low prevalence, underdiagnosis, or underreporting of symptoms by patients. The most common occurrence of all sleep disorders associated with subsequent PD occurred for restless legs syndrome, which was at least 4 times more commonly diagnosed in those with subsequent PD than in controls and was also relatively frequent (4%-6% of patients). Although restless legs syndrome is recognized as a feature of PD (it may be of heterogeneous origin^51^), it is also common in the general population. Thus far, there has been controversial evidence for an association of restless legs syndrome and subsequent PD.^11,12,52^ Among the sensory systems, hyposmia is recognized to be almost universally present in established PD and predates the diagnoses often by many years or decades.^10,53,54,55^ However, it rarely leads to subjective complaints severe enough to require medical attention. Nevertheless, we found that anosmia, the most severe form of loss of sense of smell, was more common in those with subsequent diagnosis of PD, albeit rare (<1%), in all examined time periods. We also found that hearing loss, a relatively common disorder in the general population, was more prevalent in those with subsequent diagnosis of PD than in controls, even more than 5 years before diagnosis. Although an association of hearing loss with Alzheimer disease has long been recognized,^56,57^ this has only rarely been reported for PD.^41,58^ Subjective visual complaints, which are also common in PD,^59^ were not a common feature associated with subsequent PD. Unspecified pain, another common sensory feature of PD,^60^ was present in a large number of patients before the diagnosis of PD and more common than in controls in all examined time periods as has been previously reported.^1^ To our knowledge, a new finding of this study was an association with diagnoses reflecting changes in skin sensation. Such sensations have been reported in established PD before^61,62^ but not as a prodromal feature of PD. If confirmed in future studies, this may indicate early sensory changes that reflect central changes in skin perception similar to pain but may also be linked with skin disorders as outlined subsequently. However, as the diagnostic codes used may reflect a number of different complaints, further research is needed to identify whether there is a more specific association for some of these sensory complaints.

Consistent with previous reports,^4^ results of our study suggest that risk factors such as traumatic brain injury and alcohol misuse were positively associated with a diagnosis of PD, and nicotine use was negatively associated with PD. There was also an increased OR for previous diagnoses of hypertension and hypercholesterinemia in those with subsequent diagnosis of PD, in keeping with some but not other previous reports.^26,27,28,29^ Diabetes type 2 has previously been reported to be associated with subsequent diagnosis of PD, although more and larger-scale studies were thought to be required,^31^ and diabetes type 1 has not been previously reported to be increased in patients with PD or before diagnosis. If confirmed, these associations may represent potentially modifiable risk factors for PD and may also suggest potential mechanisms contributing to the evolution of PD. Although vascular pathology may lead to development of parkinsonian syndromes not related to an underlying α-synucleinopathy, mendelian randomization and preclinical studies have suggested that diabetes is causally related to occurrence and progression of PD.^30,31,63^

### Comorbidities

We found associations of schizophrenia and bipolar disorder with a subsequent diagnosis of PD, with a 4- to 5-fold increase in risk across all time periods. Although a proportion of these cases may be due to use of dopamine antagonistic medications, which cannot always be discontinued when parkinsonism occurs, there is also increasing evidence that the use of antidopaminergics may not be the only driver of these associations^36,37^ but rather other factors such as a shared genetic background of both disorders.^36,64^ A recent study^37^ that used several approaches to investigate the association of schizophrenia with subsequent development of PD (including clinical records and diagnoses made by neurologists based on the UK Brain Bank or the Movement Disorder Society clinical criteria with follow-up over several years, the use of time limits for diagnosis and patient age, and the exclusion of patients with secondary parkinsonism) showed a clear associated increased risk of PD in those with schizophrenia, with abnormal DaTscans in those examined. Our own study, however, did not allow us to identify the medication of the cases to test this assumption further, and it is likely that at least some of the association is nevertheless secondary to the use of dopamine antagonistic medication. Similar confounding may partly contribute to the greater than 2-fold increased associated risk of epilepsy in the prediagnostic period, related to the use of the antiepileptic sodium valproate, and the less-pronounced but consistent increased rate of migraine in all prediagnostic time periods. It is also possible that patients with these diagnoses are more likely to be diagnosed with PD as they are already under neurologic or other medical follow-up care explaining some of the increase in risk.

In addition to the changes in skin sensation previously discussed, there was an association with a number of skin disorders that were examined because of their previously reported association with established or prodromal PD.^15,65^ These included not only seborrheic dermatitis, which is common in PD, but also psoriasis and dermatophytosis, reflecting fungal infection of the skin. Although the diagnostic certainty of these diagnoses is not known, these findings suggest early skin involvement, eg, through deposition of α-synuclein, which has been suggested to provide a means for early diagnosis through skin biopsy.^17,66,67^ Given the interest in the early involvement of the gastrointestinal system, with possible infectious etiology and the possible propagation of PD-related pathology through the vagal nerve, we examined associations of a number of gastrointestinal diagnoses with subsequent diagnosis of PD. We did not find a significant association with cytomegalovirus disease or infectious mononucleosis, which had been previously postulated^19,20,21^ during the observation period. However, the rarity of these diagnoses precludes firm conclusions. On the other hand, we found that gastritis, gastroesophageal reflux, gastric ulcer, and, in the most recent time period, duodenal ulcer, Crohn disease, and ulcerative colitis were associated with subsequent PD. This suggests that gastrointestinal pathology beyond constipation can occur in the prodrome of PD and may reflect early changes in gut motility, changes in constitution of gastric fluid, altered composition of the gastrointestinal microbiome, gastric infections, or other pathologies (in particular, inflammatory disorders). This may also underlie the association with osteoarthritis and seronegative arthritis, which occurred even more than 5 years before diagnosis, although misattribution of some early PD symptoms to these diagnoses cannot be excluded. Overall, it is possible that patients who present in the prodromal phase of PD receive other diagnoses related to increased medical attention. This possibility of a surveillance bias is an important consideration that has been highlighted previously^68^ and may account for some of the less-pronounced associations in the years leading up to the diagnosis of PD. Taken together with the large sample size of this study, we therefore suggest cautious interpretation in terms of etiologic inference. Nonetheless, even these associations still highlight the value of an approach based on these presentations for identifying persons at higher risk of PD. Although at present these associations do individually not allow use for clinical diagnosis or counseling, several approaches exist that use a combination of prodromal features and risk factors for research purposes,^69,70,71^ and the associations found in this study could enhance these approaches as well as support exploration of different phenotypes of PD even at the earliest stages. Further research should also explore whether associations found are particularly relevant to subgroups of patients with PD, such as those with RBD or anosmia, or whether a more generalizable, multisystem prodrome exists in the majority of patients with PD.

### Strengths and Limitations

This study had several strengths. This was a large case-control study of PD and is representative of the general population of Germany in primary care. It also included information on diagnosis of PD from general and specialist practices, independent of health care professional, providing a comprehensive data set of those with a diagnosis of PD. This extends and confirms our previously reported analysis of some of the included risk factors and prodromal features of PD in the German specialist practices.^2^

This study also had limitations, as it relied on diagnosis of PD using patient medical records, and application of diagnostic criteria was not possible. Although other electronic health care databases, such as The Health Improvement Network in the UK, have shown acceptable accuracy of primary care diagnosis of PD using a single diagnostic code,^1^ albeit with slightly higher incidence rates,^72^ no validation study is available in this data source. The diagnostic codes used for prodromal features and risk factors may also not always be accurate or precise, given that the medical records used were based on a routine care database. These diagnostic limitations should be taken into account as detailed in the discussion. We were also not able to access information on medication and tried to interpret findings cautiously, where a suspected medication-induced effect is possible. However, equally unrecognized medication effects may not be acknowledged, eg, for medications used to treat gastritis or gastroesophageal reflux. Furthermore, the database only includes diagnoses made according to *ICD-10* codes. More subtle symptoms or features are likely to have been underrecognized. It is also important to note that secondary analysis of claims data is not meant to confirm, but rather to generate, hypotheses on potential associations that can be tested in subsequent primary studies.

## Conclusions

Given the size and study period, we believe that this case-control study has generated valuable hypotheses on the associations found between PD and certain risk factors, comorbidities, and prodromal symptoms in a representative population. These associations may reflect possible early extrastriatal and extracerebral pathology of PD due to shared genetic risk with PD, medication exposure, or direct causation, or represent pathophysiologically relevant factors contributing to the pathogenesis of PD. Subtle associations require future testing in prospective controlled studies.

## Notes

Supplement. **eTable 1.** Prodromal Features, Risk Factors, or Comorbidities and Corresponding *ICD* Codes **eTable 2.** Odds Ratios and 95% CIs Adjusted for Multiple Comparisons of Prodromal Features, Risk Factors, and Comorbidities in Cases Compared With Controls in the Year Before Index Date and in the Time Periods 2 to 4 Years and 5 to 10 Years Before the Index Date **eTable 3.** Prodromal Features, Risk Factors, or Comorbidities in Cases and Controls in the Year Before Index Date and in the Time Periods 2 to 4 Years and 5 to 10 Years Before the Index Date **eFigure.** Prevalence of Additional Presentations Associated With PD by Year Before Diagnosis Compared With Controls
