# Supplementary material for: Widening the Spectrum of Risk Factors, Comorbidities, and Prodromal Features of Parkinson Disease
Source: JAMA Neurol. 2022 Nov 7;80(2):161–71. doi: 10.1001/jamaneurol.2022.3902 (PMC9641600; doi:10.1001/jamaneurol.2022.3902)
Supplement: Supplement. — eTable 1. Prodromal Features, Risk Factors, or Comorbidities and Corresponding ICD Codes eTable 2. Odds Ratios and 95% CIs Adjusted for Multiple Comparisons of Prodromal Features, Risk Factors, and Comorbidities in Cases Compared With Controls in the Year Before Index Date and in the Time Periods 2 to 4 Years and 5 to 10 Years Before the Index Date eTable 3. Prodromal Features, Risk Factors, or Comorbidities in Cases and Controls in the Year Before Index Date and in the Time Periods 2 to 4 Years and 5 to 10 Years Before the Index Date eFigure. Prevalence of Additional Presentations Associated With PD by Year Before Diagnosis Compared With Controls [file jamaneurol-e223902-s001.pdf]

# Supplementary Online Content

Schrag A, Bohlken J, Dammertz L, et al. Widening the spectrum of risk factors, comorbidities, and prodromal features of Parkinson disease. *JAMA Neurol*. Published online November 7, 2022. doi:10.1001/jamaneurol.2022.3902

**eTable 1.** Prodromal Features, Risk Factors, or Comorbidities and Corresponding *ICD* Codes

**eTable 2.** Odds Ratios and 95% CIs Adjusted for Multiple Comparisons of Prodromal Features, Risk Factors, and Comorbidities in Cases Compared With Controls in the Year Before Index Date and in the Time Periods 2 to 4 Years and 5 to 10 Years Before the Index Date

**eTable 3.** Prodromal Features, Risk Factors, or Comorbidities in Cases and Controls in the Year Before Index Date and in the Time Periods 2 to 4 Years and 5 to 10 Years Before the Index Date

**eFigure.** Prevalence of Additional Presentations Associated With PD by Year Before Diagnosis Compared With Controls

This supplementary material has been provided by the authors to give readers additional information about their work.

© 2022 Schrag A et al. *JAMA Neurology.*

**eTable 1.** Prodromal Features, Risk Factors, or Comorbidities and Corresponding *ICD* Codes

| Symptoms | ICD-10 codes |
|---|---|
| **Motor dysfunction** | |
| Gait impairment | R26 |
| Neck pain | M54.2 |
| Shoulder pain | M25.51 |
| Stiffness of joints | M25.6 |
| Tremor | G25.0, G25.1, G25.2, R25.1 |
| **Psychiatric presentations** | |
| Anxiety | F41.0, F41.1, F41.9 |
| Depression | F32, F33 |
| Memory problems | F06.7, R41.3, R41.8 |
| **Impairment of sensory experiences** | |
| Hearing loss | H91.2, H91.9 |
| Anosmia | R43.0 |
| Changes of skin sensation | R20.0, R20.1, R20.2, R20.3 |
| Subjective visual disturbance | H53.1 |
| Non-specific pain | R52.1, R52.2, R52.9 |
| **Sleep disorders** | |
| Restless legs syndrome | G25.80, G25.81 |
| Hypersomnia | F51.1 with G47.1 |
| Insomnia | F51.0 with G47.0 |
| Other sleep disorders | F51.2, F51.8, F51.9, G47.2, G47.9 |
| Parasomnia | F51.3, F51.4, F51.5, G47.8 |
| Sleep apnea | G47.3 |
| **Skin diseases** | |
| Dermatophytosis | B35 |
| Psoriasis | L40 |
| Seborrheic dermatitis | L21 |
| **Autonomic dysfunction and fatigue** | |
| Constipation | K59.0 |
| Dizziness | R42 |
| Fatigue | G93.3 |
| Neurogenic bladder | N31 |
| Orthostasis | I95, R03.1 |
| Sexual dysfunction | F52.0, F52.1, F52.2, F52.8, F52.9 |
| **Risk factors and comorbidities** | |
| **Risk factors** | |
| Alcohol abuse | F10.1, F10.2 |
| Nicotine abuse | F17.1, F17.2 |
| Traumatic brain injury | S06.0, S06.1, S06.2, S06.3 |
| **Metabolic diseases and vascular risk factors** | |

© 2022 Schrag A et al. *JAMA Neurology.*

| | |
|---|---|
| Diabetes mellitus type 1 | E10 |
| Diabetes mellitus type 2 | E11 |
| Hypercholesterinemia | E78 |
| Hypertension | I10 |
| **Infectious diseases** | |
| Cytomegaloviral disease | B25 |
| Infectious mononucleosis | B27 |
| **Gastrointestinal disorders** | |
| Duodenal ulcer | K26 |
| Gastric ulcer | K25 |
| Gastritis | K29 |
| Gastro-oesophageal reflux disease | K21 |
| Gastrojejunal ulcer | K28 |
| Peptic ulcer | K27 |
| Crohn's disease | K50 |
| Ulcerative colitis | K51 |
| **Other comorbidities** | |
| Osteoarthritis | M15, M16, M17, M18, M19 |
| Seropositive inflammatory arthritis | M05 |
| Other inflammatory arthritis | M06 |
| Bipolar disorder | F31 |
| Schizophrenia | F20 |
| Epilepsy | G40 |
| Migraine | G43 |

© 2022 Schrag A et al. *JAMA Neurology.*

**eTable 2.** Odds Ratios and 95% CIs Adjusted for Multiple Comparisons of Prodromal Features, Risk Factors, and Comorbidities in Cases Compared With Controls in the Year Before Index Date and in the Time Periods 2 to 4 Years and 5 to 10 Years Before the Index Date

| | Within 1st year before index date | 2 to 4 years before index date | 5 to 10 years before index date |
|---|---|---|---|
| **Symptoms** | | | |
| **Motor dysfunction** | | | |
| Gait impairment | 1.96 (1.88-2.03) | 1.64 (1.57-1.71) | 1.35 (1.25-1.46) |
| Neck pain | 1.22 (1.16-1.29) | 1.16 (1.11-1.21) | 1.11 (1.06-1.17) |
| Shoulder pain | 1.26 (1.09-1.46) | 1.17 (1.05-1.30) | 1.04 (0.91-1.18) |
| Stiffness of joints | 1.42 (1.17-1.73) | 1.26 (1.07-1.48) | 1.23 (1.01-1.49) |
| Tremor | 14.94 (13.66-16.33) | 7.13 (6.53-7.77) | 4.49 (3.98-5.06) |
| **Psychiatric presentations** | | | |
| Anxiety | 1.82 (1.71-1.93) | 1.66 (1.58-1.75) | 1.47 (1.38-1.57) |
| Depression | 2.04 (1.98-2.10) | 1.82 (1.77-1.87) | 1.57 (1.52-1.62) |
| Memory problems | 1.77 (1.63-1.92) | 1.59 (1.46-1.73) | 1.49 (1.29-1.71) |
| **Impairment of sensory organs** | | | |
| Hearing loss | 1.17 (1.10-1.24) | 1.17 (1.11-1.23) | 1.12 (1.05-1.19) |
| Anosmia | 2.45 (1.71-3.51) | 2.10 (1.52-2.91) | 2.00 (1.35-2.97) |
| Impairment of skin sensation | 1.69 (1.47-1.94) | 1.26 (1.13-1.39) | 1.21 (1.07-1.37) |
| Subjective visual disturbance | 1.30 (0.86-1.99) | 1.22 (0.94-1.58) | 1.27 (0.95-1.69) |
| Non-specific pain | 1.53 (1.47-1.58) | 1.44 (1.40-1.50) | 1.36 (1.29-1.43) |
| **Sleep disorders** | | | |
| Restless legs syndrome | 4.74 (4.41-5.10) | 4.24 (3.95-4.55) | 3.73 (3.39-4.09) |
| Hypersomnia | 2.64 (1.35-5.18) | 2.58 (1.43-4.64) | 1.90 (0.84-4.29) |
| Insomnia | 1.49 (1.38-1.61) | 1.42 (1.33-1.52) | 1.32 (1.21-1.44) |
| Other sleep disorders | 1.46 (1.39-1.54) | 1.40 (1.34-1.47) | 1.32 (1.25-1.40) |
| Parasomnia | 1.77 (1.49-2.10) | 1.53 (1.32-1.77) | 1.48 (1.23-1.78) |
| Sleep apnea | 1.47 (1.38-1.55) | 1.41 (1.33-1.50) | 1.35 (1.26-1.45) |
| **Skin diseases** | | | |
| Dermatophytosis | 1.30 (1.20-1.40) | 1.25 (1.18-1.33) | 1.19 (1.11-1.27) |
| Psoriasis | 1.11 (1.04-1.20) | 1.11 (1.04-1.19) | 1.09 (1.01-1.18) |
| Seborrheic dermatitis | 1.42 (1.20-1.67) | 1.32 (1.15-1.50) | 1.22 (1.04-1.42) |
| **Autonomic dysfunction and fatigue** | | | |
| Constipation | 2.00 (1.88-2.12) | 1.86 (1.76-1.96) | 1.65 (1.53-1.77) |
| Dizziness | 1.80 (1.74-1.87) | 1.56 (1.51-1.61) | 1.33 (1.28-1.40) |
| Fatigue | 1.72 (1.61-1.83) | 1.38 (1.31-1.45) | 1.17 (1.10-1.25) |
| Neurogenic bladder | 1.77 (1.55-2.02) | 1.67 (1.47-1.91) | 1.56 (1.29-1.88) |
| Orthostasis | 1.47 (1.36-1.60) | 1.37 (1.28-1.46) | 1.24 (1.14-1.35) |
| Sexual dysfunction | 1.19 (1.08-1.32) | 1.21 (1.11-1.31) | 1.21 (1.10-1.34) |
| **Risk factors and comorbidities** | | | |
| **Risk factors** | | | |
| Alcohol abuse | 1.32 (1.21-1.44) | 1.28 (1.18-1.39) | 1.23 (1.11-1.36) |
| Nicotine abuse | 0.93 (0.87-0.99) | 0.93 (0.87-0.99) | 0.91 (0.84-0.98) |
| Traumatic brain injury | 2.05 (1.55-2.70) | 1.44 (1.16-1.79) | 1.40 (1.07-1.84) |
| **Metabolic diseases and vascular risk factors** | | | |
| Diabetes mellitus type 1 | 1.35 (1.22-1.49) | 1.33 (1.22-1.45) | 1.30 (1.17-1.45) |
| Diabetes mellitus type 2 | 1.25 (1.22-1.29) | 1.24 (1.21-1.28) | 1.23 (1.19-1.27) |
| Hypercholesterinaemia | 1.09 (1.07-1.12) | 1.09 (1.07-1.11) | 1.06 (1.04-1.09) |
| Hypertension | 1.24 (1.22-1.26) | 1.22 (1.20-1.24) | 1.17 (1.15-1.20) |
| **Infectious diseases** | | | |
| Cytomegaloviral disease | 1.07 (0.49-2.34) | 1.10 (0.60-2.04) | 0.84 (0.42-1.66) |

© 2022 Schrag A et al. *JAMA Neurology.*

| | | | |
|---|---|---|---|
| Infectious mononucleosis | 1.78 (0.93-3.41) | 1.46 (0.87-2.45) | 1.53 (0.82-2.85) |
| **Gastrointestinal disorders** | | | |
| Duodenal ulcer | 1.12 (0.97-1.31) | 1.13 (0.99-1.29) | 1.18 (1.01-1.37) |
| Gastric ulcer | 1.29 (1.14-1.46) | 1.25 (1.12-1.39) | 1.24 (1.09-1.42) |
| Gastritis | 1.32 (1.27-1.37) | 1.27 (1.23-1.32) | 1.23 (1.19-1.28) |
| Gastro-oesophageal reflux disease | 1.27 (1.23-1.32) | 1.26 (1.22-1.30) | 1.24 (1.19-1.29) |
| Gastrojejunal ulcer | 1.32 (0.78-2.22) | 1.32 (0.83-2.09) | 1.10 (0.62-1.95) |
| Peptic ulcer | 1.43 (0.96-2.15) | 1.35 (0.96-1.91) | 1.20 (0.80-1.81) |
| Crohn's disease | 1.31 (1.07-1.61) | 1.20 (0.98-1.46) | 1.15 (0.91-1.45) |
| Ulcerative colitis | 1.26 (1.07-1.47) | 1.22 (1.06-1.42) | 1.16 (0.97-1.38) |
| **Other comorbidities** | | | |
| Osteoarthritis | 1.20 (1.17-1.23) | 1.18 (1.15-1.21) | 1.13 (1.09-1.16) |
| Seropositive inflammatory arthritis | 1.21 (0.99-1.48) | 1.15 (0.95-1.39) | 1.10 (0.86-1.40) |
| Other inflammatory arthritis | 1.19 (1.10-1.29) | 1.19 (1.11-1.29) | 1.15 (1.05-1.27) |
| Bipolar disorder | 4.09 (3.22-5.21) | 3.95 (3.13-4.98) | 3.80 (2.82-5.14) |
| Schizophrenia | 4.69 (3.99-5.50) | 4.29 (3.68-5.00) | 4.00 (3.31-4.85) |
| Epilepsy | 2.44 (2.24-2.65) | 2.20 (2.02-2.40) | 2.02 (1.81-2.26) |
| Migraine | 1.24 (1.15-1.34) | 1.21 (1.13-1.29) | 1.17 (1.09-1.27) |

© 2022 Schrag A et al. *JAMA Neurology.*

**eTable 3.** Prodromal Features, Risk Factors, or Comorbidities in Cases and Controls in the Year Before Index Date and in the Time Periods 2 to 4 Years and 5 to 10 Years Before the Index Date

| | ≤1st year before index date | | 2 to 4 years before index date | | 5 to 10 years before index date | |
|---|---|---|---|---|---|---|
| | **Cases (n=138,345)** | **Controls (n=276,690)** | **Cases (n=138,345)** | **Controls (n=276,690)** | **Cases (n=106,957)** | **Controls (n=213,914)** |
| **Symptoms** | | | | | | |
| **Motor dysfunction** | | | | | | |
| Gait impairment | 18274 (13.2%) | 19980 (7.2%) | 13410 (9.7%) | 17007 (6.1%) | 3417 (3.2%) | 5103 (2.4%) |
| Neck pain | 10139 (7.3%) | 16828 (6.1%) | 15719 (11.4%) | 27626 (10.0%) | 11647 (10.9%) | 21146 (9.9%) |
| Shoulder pain | 1264 (0.9%) | 2004 (0.7%) | 2520 (1.8%) | 4323 (1.6%) | 1537 (1.4%) | 2958 (1.4%) |
| Stiffness of joints | 759 (0.5%) | 1069 (0.4%) | 1033 (0.7%) | 1642 (0.6%) | 688 (0.6%) | 1124 (0.5%) |
| Tremor | 12888 (9.3%) | 1890 (0.7%) | 7688 (5.6%) | 2266 (0.8%) | 2933 (2.7%) | 1335 (0.6%) |
| **Psychiatric presentations** | | | | | | |
| Anxiety | 7257 (5.2%) | 8187 (3.0%) | 8823 (6.4%) | 10900 (3.9%) | 5434 (5.1%) | 7498 (3.5%) |
| Depression | 33661 (24.3%) | 37696 (13.6%) | 36100 (26.1%) | 45053 (16.3%) | 22589 (21.1%) | 31170 (14.6%) |
| Memory problems | 3823 (2.8%) | 4369 (1.6%) | 3287 (2.4%) | 4171 (1.5%) | 1135 (1.1%) | 1532 (0.7%) |
| **Impairment of sensory organs** | | | | | | |
| Hearing loss | 5542 (4.0%) | 9568 (3.5%) | 7710 (5.6%) | 13295 (4.8%) | 5406 (5.1%) | 9742 (4.6%) |
| Anosmia | 223 (0.2%) | 182 (0.1%) | 250 (0.2%) | 238 (0.1%) | 167 (0.2%) | 167 (0.1%) |
| Change of skin sensation | 1291 (0.9%) | 1534 (0.6%) | 1999 (1.4%) | 3193 (1.2%) | 1367 (1.3%) | 2266 (1.1%) |
| Subjective visual disturbance | 120 (0.1%) | 184 (0.1%) | 302 (0.2%) | 497 (0.2%) | 248 (0.2%) | 392 (0.2%) |
| Non-specific pain | 20104 (14.5%) | 27740 (10.0%) | 20171 (14.6%) | 29233 (10.6%) | 9554 (8.9%) | 14398 (6.7%) |
| **Sleep disorders** | | | | | | |
| Restless legs syndrome | 8315 (6.0%) | 3682 (1.3%) | 7968 (5.8%) | 3929 (1.4%) | 4147 (3.9%) | 2291 (1.1%) |
| Hypersomnia | 66 (0.0%) | 50 (0.0%) | 85 (0.1%) | 66 (0.0%) | 38 (0.0%) | 40 (0.0%) |
| Insomnia | 3712 (2.7%) | 5024 (1.8%) | 4977 (3.6%) | 7096 (2.6%) | 3061 (2.9%) | 4670 (2.2%) |
| Other sleep disorders | 7946 (5.7%) | 11071 (4.0%) | 10790 (7.8%) | 15725 (5.7%) | 6939 (6.5%) | 10696 (5.0%) |
| Parasomnia | 844 (0.6%) | 957 (0.3%) | 1098 (0.8%) | 1440 (0.5%) | 666 (0.6%) | 900 (0.4%) |
| Sleep apnea | 6831 (4.9%) | 9474 (3.4%) | 7001 (5.1%) | 10062 (3.6%) | 4252 (4.0%) | 6357 (3.0%) |
| **Skin diseases** | | | | | | |
| Dermatophytosis | 3868 (2.8%) | 5995 (2.2%) | 6549 (4.7%) | 10556 (3.8%) | 4696 (4.4%) | 7947 (3.7%) |
| Psoriasis | 4087 (3.0%) | 7357 (2.7%) | 4619 (3.3%) | 8345 (3.0%) | 3315 (3.1%) | 6112 (2.9%) |
| Seborrheic dermatitis | 800 (0.6%) | 1132 (0.4%) | 1233 (0.9%) | 1878 (0.7%) | 861 (0.8%) | 1418 (0.7%) |
| **Autonomic dysfunction and fatigue** | | | | | | |
| Constipation | 6963 (5.0%) | 7158 (2.6%) | 9587 (6.9%) | 10660 (3.9%) | 4766 (4.5%) | 5889 (2.8%) |
| Dizziness | 19152 (13.8%) | 22629 (8.2%) | 22868 (16.5%) | 31161 (11.3%) | 11711 (10.9%) | 18049 (8.4%) |
| Fatigue | 6247 (4.5%) | 7423 (2.7%) | 8863 (6.4%) | 13120 (4.7%) | 4732 (4.4%) | 8131 (3.8%) |
| Neurogenic bladder | 1376 (1.0%) | 1561 (0.6%) | 1391 (1.0%) | 1669 (0.6%) | 658 (0.6%) | 848 (0.4%) |
| Orthostasis | 3405 (2.5%) | 4665 (1.7%) | 4848 (3.5%) | 7168 (2.6%) | 3130 (2.9%) | 5083 (2.4%) |

© 2022 Schrag A et al. *JAMA Neurology.*

| | | | | | | |
|---|---|---|---|---|---|---|
| Sexual dysfunction | 2248 (1.6%) | 3774 (1.4%) | 2943 (2.1%) | 4891 (1.8%) | 2246 (2.1%) | 3717 (1.7%) |
| **Risk factors and comorbidities** | | | | | | |
| **Risk factors** | | | | | | |
| Alcohol abuse | 2894 (2.1%) | 4392 (1.6%) | 3155 (2.3%) | 4962 (1.8%) | 2037 (1.9%) | 3336 (1.6%) |
| Nicotine abuse | 4627 (3.3%) | 9917 (3.6%) | 5303 (3.8%) | 11407 (4.1%) | 3471 (3.2%) | 7635 (3.6%) |
| Traumatic brain injury | 335 (0.2%) | 328 (0.1%) | 479 (0.3%) | 665 (0.2%) | 301 (0.3%) | 429 (0.2%) |
| **Metabolic diseases and vascular risk factors** | | | | | | |
| Diabetes mellitus type 1 | 2140 (1.5%) | 3194 (1.2%) | 2885 (2.1%) | 4357 (1.6%) | 1932 (1.8%) | 2977 (1.4%) |
| Diabetes mellitus type 2 | 40165 (29.0%) | 68098 (24.6%) | 40485 (29.3%) | 69045 (25.0%) | 26716 (25.0%) | 45503 (21.3%) |
| Hypercholesterinemia | 59463 (43.0%) | 112979 (40.8%) | 65653 (47.5%) | 125478 (45.3%) | 47966 (44.8%) | 92783 (43.4%) |
| Hypertension | 93814 (67.8%) | 174135 (62.9%) | 97145 (70.2%) | 182316 (65.9%) | 69182 (64.7%) | 130454 (61.0%) |
| **Infectious diseases** | | | | | | |
| Cytomegaloviral disease | 32 (0.0%) | 60 (0.0%) | 53 (0.0%) | 96 (0.0%) | 39 (0.0%) | 93 (0.0%) |
| Infectious mononucleosis | 58 (0.0%) | 65 (0.0%) | 83 (0.1%) | 114 (0.0%) | 59 (0.1%) | 77 (0.0%) |
| **Gastrointestinal disorders** | | | | | | |
| Duodenal ulcer | 874 (0.6%) | 1555 (0.6%) | 1136 (0.8%) | 2018 (0.7%) | 851 (0.8%) | 1449 (0.7%) |
| Gastric ulcer | 1372 (1.0%) | 2132 (0.8%) | 1821 (1.3%) | 2932 (1.1%) | 1219 (1.1%) | 1966 (0.9%) |
| Gastritis | 16013 (11.6%) | 24934 (9.0%) | 21948 (15.9%) | 35694 (12.9%) | 15725 (14.7%) | 26228 (12.3%) |
| Gastro-oesophageal reflux disease | 20262 (14.6%) | 32848 (11.9%) | 23307 (16.8%) | 38259 (13.8%) | 16145 (15.1%) | 26796 (12.5%) |
| Gastrojejunal ulcer | 79 (0.1%) | 120 (0.0%) | 100 (0.1%) | 152 (0.1%) | 61 (0.1%) | 111 (0.1%) |
| Peptic ulcer | 137 (0.1%) | 191 (0.1%) | 181 (0.1%) | 268 (0.1%) | 123 (0.1%) | 205 (0.1%) |
| Crohn's disease | 500 (0.4%) | 764 (0.3%) | 526 (0.4%) | 879 (0.3%) | 370 (0.4%) | 646 (0.3%) |
| Ulcerative colitis | 856 (0.6%) | 1363 (0.5%) | 971 (0.7%) | 1590 (0.6%) | 646 (0.6%) | 1119 (0.5%) |
| **Other comorbidities** | | | | | | |
| Osteoarthritis | 41339 (29.9%) | 72653 (26.3%) | 46213 (33.4%) | 82422 (29.8%) | 30589 (28.6%) | 56149 (26.2%) |
| Seropositive inflammatory arthritis | 674 (0.5%) | 1114 (0.4%) | 736 (0.5%) | 1279 (0.5%) | 443 (0.4%) | 806 (0.4%) |
| Other inflammatory arthritis | 4171 (3.0%) | 7023 (2.5%) | 4690 (3.4%) | 7901 (2.9%) | 3088 (2.9%) | 5372 (2.5%) |
| Bipolar disorder | 862 (0.6%) | 423 (0.2%) | 912 (0.7%) | 464 (0.2%) | 535 (0.5%) | 282 (0.1%) |
| Schizophrenia | 1678 (1.2%) | 723 (0.3%) | 1716 (1.2%) | 808 (0.3%) | 1055 (1.0%) | 531 (0.2%) |
| Epilepsy | 4138 (3.0%) | 3452 (1.2%) | 3849 (2.8%) | 3549 (1.3%) | 2151 (2.0%) | 2148 (1.0%) |
| Migraine | 3744 (2.7%) | 6070 (2.2%) | 4468 (3.2%) | 7439 (2.7%) | 3587 (3.4%) | 6147 (2.9%) |

© 2022 Schrag A et al. *JAMA Neurology.*

**eFigure.** Prevalence of Additional Presentations Associated With PD by Year Before Diagnosis Compared With Controls

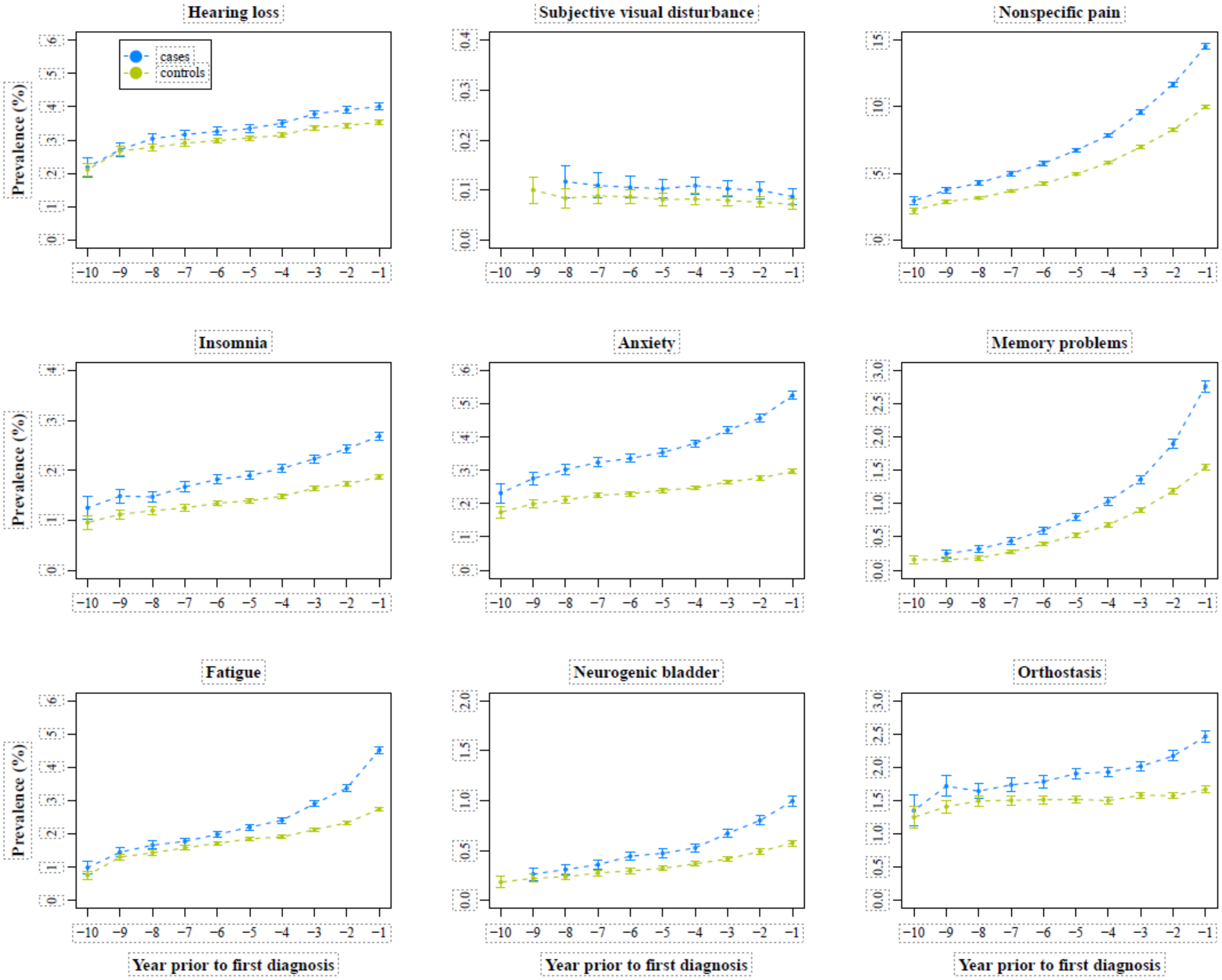


© 2022 Schrag A et al. *JAMA Neurology.*

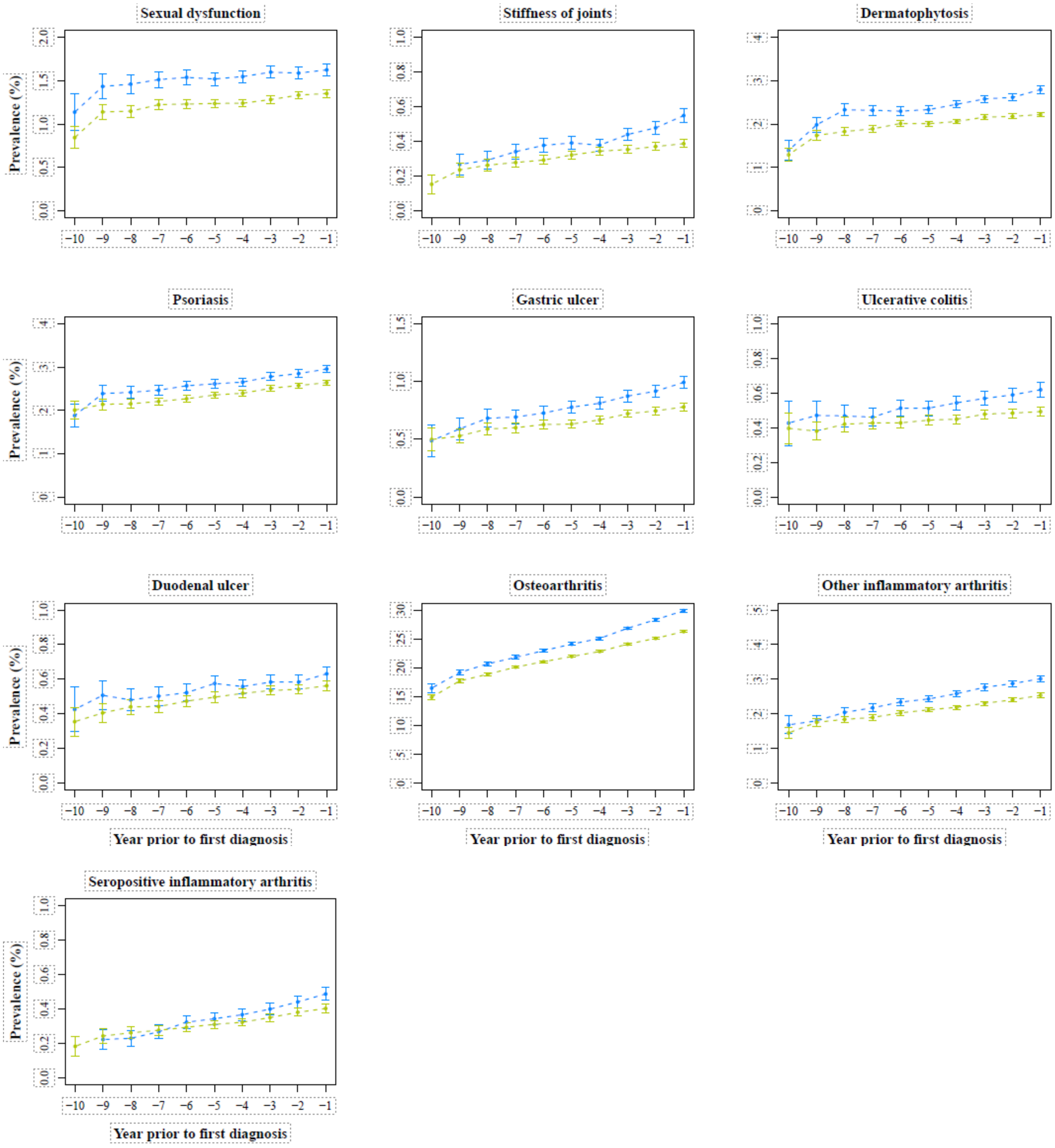


Supplementary Figure 1 Prevalence (%) with 95% CI error bars for each year before diagnosis of Parkinson's disease. Part A Hearing Loss, Part B Subjective visual impairment, Part C Non-specific pain, Part D Insomnia, Part E Anxiety, Part F Memory problems, Part G Fatigue, Part H Neurogenic bladder, Orthostasis, Part I Sexual dysfunction, Part J Stiffness of joints, Part K Dermatophytosis, Part L Psoriasis, Gastric ulcer, Part M Ulcerative colitis, Part N Duodenal Ulcer, Part O Osteoarthristis, Part P Other inflammatory arthritis, Part Q Seropositive inflammatory arthritis

© 2022 Schrag A et al. *JAMA Neurology.*
